# SG-APSIC1081: Healthcare cost of antibiotic resistant infections: A hospital-based study in Vietnam

**DOI:** 10.1017/ash.2023.80

**Published:** 2023-03-16

**Authors:** Thi LanPham, Lan Pham

**Affiliations:** University Medical Center, Ho Chi Minh City, Vietnam; University Medical Center, Ho Chi Minh City, Vietnam

## Abstract

**Objectives:** Antimicrobial resistance is a serious threat to health and economic well-being worldwide. The impact of antibiotic-resistant infections is reflected by higher mortality, increased lengths of hospital stay (LOS), and increased healthcare costs. We analyzed the direct healthcare costs attributable to treating patients infected with methicillin-resistant *Staphylococcus aureus* (MRSA) and carbapenem-resistant Enterobacterales (CRE) in a tertiary-care referral hospital in Vietnam. **Methods:** A retrospective descriptive cross-sectional study was conducted in an intensive care unit (ICU) in the University Medical Center in Ho Chi Minh City from June 2018 to September 2019. Participants were ICU patients diagnosed with either MRSA or CRE infection, and patients infected with non–multidrug-resistant organisms (non-MDROs) were used as the comparison group. Medical records were obtained to collect data on medical services expenditures such as medications, diagnostic testing, medical procedures, and hospital rooms. Statistical significance was determined using the Mann-Whitney and Kruskal-Wallis tests for comparing the average costs and the χ^2^ test for comparing the proportions. **Results:** In total, 227 patients, including 37 MRSA-infected patients, 97 CRE-infected patients, and 93 non–MDRO-infected patients, were included in the study. The additional average healthcare costs for a treatment episode of a CRE infection case (367.7 million VND or ~US $16,000) and of a MRSA infection case (139.1 million VND or ~US $6,043) were 3.8 times higher (*P* < .001) and 1.5 times higher (*P* < .001), respectively, than the average cost for a non-MDRO case (94.8 million VND or ~US $4,121). Resource use for a CRE infection was higher than that for MRSA infection, with longer antibiotic treatment (13.4 additional days), greater LOS (15.8 additional days), and higher costs (additional 228.6 million VND or ~US $9,939). **Conclusions:** Multidrug-resistant infections create a heavy economic burden in a low- to middle-income country like Vietnam. The elevated cost was mainly due to longer antibiotic treatment and increased LOS.

